# Evaluation of performance indicators of a national breast cancer screening program in Casablanca, Morocco

**DOI:** 10.1186/s12885-025-14017-y

**Published:** 2025-04-04

**Authors:** Melissa M. Carvalho, Aissatou Bah, Sofia Azrib, Robert A. Hiatt, Saber Boutayeb, Lahcen Belyamani, Amr S. Soliman, Mohamed Khalis

**Affiliations:** 1 Institute for Global Health Sciences, University of California San Francisco, San Francisco, CA USA; 2 Mohammed VI International School of Public Health, Mohammed VI University of Sciences and Health, Casablanca, Morocco; 3 Public Health Service, Region of Casablanca-Settat, Morocco Ministry of Health, Casablanca, Morocco; 4 Department of Epidemiology, University of California San Francisco, San Francisco, CA USA; 5 Department of Public Health and Clinical Research, Mohammed VI Center for Research and Innovation, Rabat, Morocco; 6 Faculty of Medicine, Mohammed VI University of Sciences and Health, Casablanca, Morocco; 7 Department of Community Health and Social Medicine, City University of New York School of Medicine, The City College of New York, New York, NY USA; 8 Higher Institute of Nursing Professions and Health Techniques, Rabat, Ministry of Health and Social Protection, Rabat, Morocco; 9 Box 1302, University of California San Francisco, 550 16th St, San Francisco, CA 94158 USA

**Keywords:** Breast cancer screening, Cancer prevention & control, Global cancer, Monitoring & evaluation of global health programs

## Abstract

**Background:**

Morocco’s national breast cancer screening program (NBCSP) was launched in 2010 in response to rising breast cancer incidence. The program comprises clinical breast examination (CBE) and diagnostic mammography and aims to improve early diagnosis and breast cancer management. This study evaluated key performance indicators of the NBCSP for the Casablanca-Settat region from 2018 to 2021.

**Methods:**

Aggregated regional data on screening and diagnostic activities under the NBCSP were extracted from a health information system. Annual screening coverage, participation, CBE-positivity, and breast cancer detection rates for the region were then calculated. Screening numbers and CBE positivity rates were compared by year and sub-region using one-way ANOVA and Bonferroni multiple-comparison tests. Trends in breast cancer screenings were also compared using Pearson’s correlation coefficients and statistical trend tests.

**Results:**

From 2018 to 2021, a total of 846,692 women in the Casablanca-Settat region were screened under the NBCSP, and 21,476 referred for a positive CBE to a designated secondary referral center for diagnostic mammography. Annual screening coverage rates of eligible women ranged from 10.4 to 28.8% during the study period. Only two out of nine administrative sub-regions in Casablanca-Settat achieved a desired 40% screening coverage threshold. Overall, annual participation among the target population from 2018 to 2021 decreased by 44.8%. Annual CBE positivity rates remained stable between 2.2 and 2.7%, though notable variations were observed at the sub-regional level. Over one-third (36.4%, *n* = 7,808) of CBE-positive women sought consultations at designated secondary referral centers in the region. Compliance to further diagnostic testing at these centers increased overtime, from 24.2% in 2018 to 61.9% in 2021. Breast cancer detection rates in the region from 2018 to 2021 were: 0.7, 0.8, 1.0, to 1.1 per 1000 women screened, respectively.

**Conclusions:**

The NBCSP fell short of achieving its desired performance benchmarks in the Casablanca-Settat region from 2018 to 2021, notably in screening coverage, participation, and CBE positivity rates. Annual breast cancer detection rates under the program also remained low. Additional interventions are needed to increase screening participation, standardize CBE training, and establish linkages between health facilities to limit the underestimation of breast cancer under the NBCSP.

## Background

Rising rates of cancer in the Middle East and North Africa have led the World Health Organization (WHO) to recommend that countries in the region develop sustainable cancer control strategies to address malignancies that most affect their populations [1]. In Morocco, breast cancer is the most commonly diagnosed cancer and leading cause of cancer-related deaths among women. In 2020, it represented 39% of new female cancer cases and accounted for 12.2% of female cancer deaths in the country [2]. Population-based cancer registry data in the Greater Casablanca area and city of Rabat also suggest that a majority of breast cancers (69%) are diagnosed at late stages [2–3]. Morocco, like other emerging economies, has seen its breast cancer incidence rise in recent decades, likely driven by improvements in reporting systems and greater exposure to risk factors associated with increased globalization and economic and social development [4].

In response to this rising breast cancer burden, the Moroccan government, through its National Cancer Prevention and Control Plan, facilitated the implementation of a national breast cancer screening program (NBCSP) in 2010 [5]. The NBCSP aims to improve the management of breast cancer patients in Morocco through an established system for screening, early diagnosis, and treatment [6]. The NBCSP is opportunistic in nature and is based on clinical breast examination (CBE) supplemented by diagnostic mammography. CBE is considered to be a promising approach to achieve downstaging in low and middle-income countries (LMICs) when adequate diagnostic and therapeutic facilities are present [7]. Moreover, its cost-effectiveness has been demonstrated in simulation modeling studies of low-resource settings [8].

The WHO recommends periodically assessing the effectiveness of screening and early detection programs [9]. Monitoring and evaluating breast cancer screening programs at regular intervals via performance indicators is essential to ensuring they meet quality standards and deliver expected benefits [10]. In Morocco, two prior studies evaluated early performance indicators for the NBCSP in Temara city and the Meknes-Tafilalt region between 2009 and 2014. Both studies found moderate to low screening coverage for the NBCSP and low breast cancer detection rates [11, 12]. A comprehensive evaluation of the program was also conducted at the national level by the International Agency for Research on Cancer in 2017 [13]. No study, however, exclusively focused on the program’s performance in the Casablanca-Settat region, which serves as the economic center of the country. This region is more urban and socio-demographically diverse than the population of Morocco as a whole [14, 15]. Consequently, an evaluation of the NBCSP in this setting fills an important knowledge gap on regional differences in screening participation and breast cancer detection rates under the NBCSP. The purpose of this study was to evaluate key performance indicators of the screening program in the Casablanca-Settat region over a four-year period and compare them against national quality standards established by the Moroccan Ministry of health (MOH).

## Methods

### Study design and setting

**Fig. 1:**
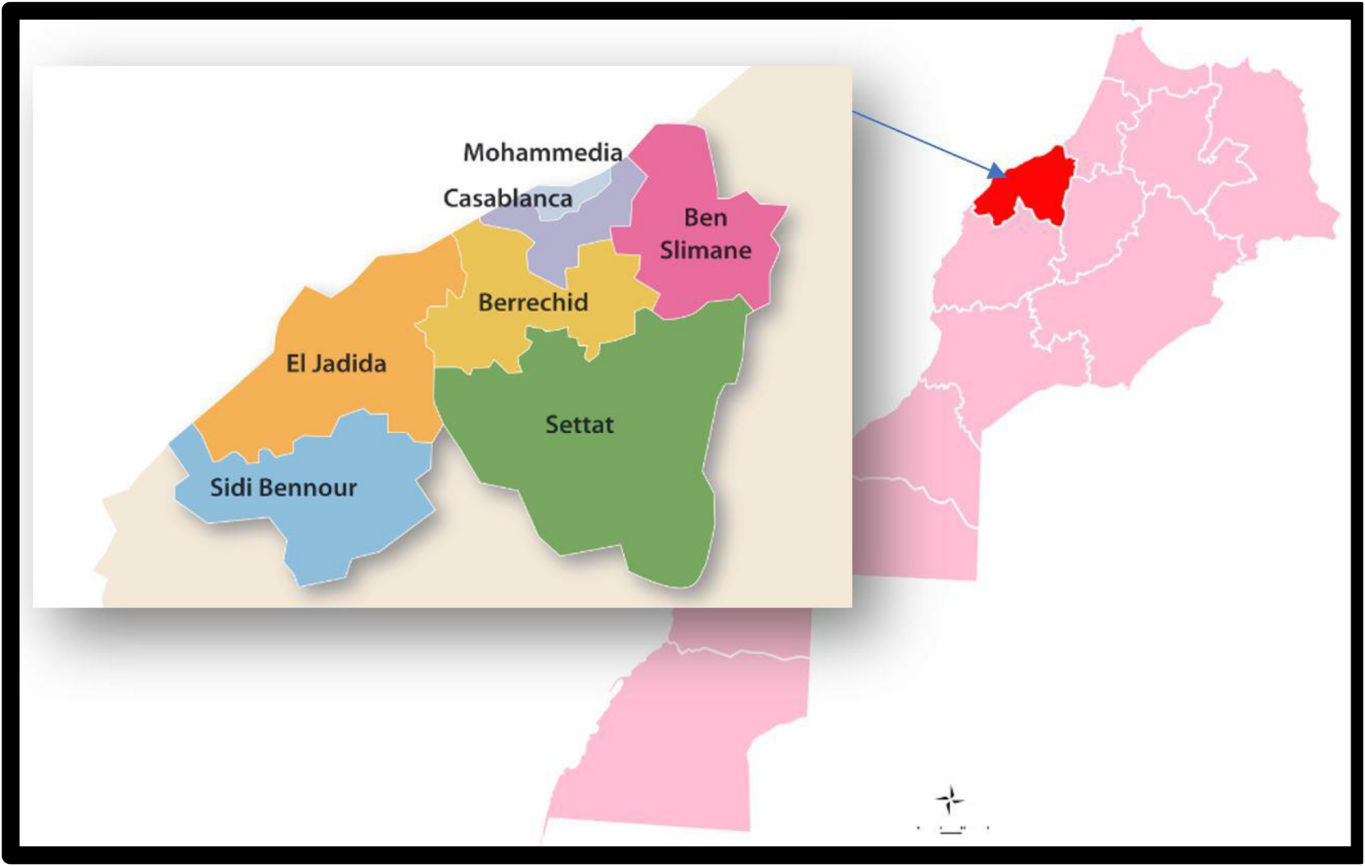
Map of the provinces and prefectures of the Casablanca-Settat region. Source: Morocco Ministry of Equipment and Water (2022)

We conducted a retrospective evaluative study of the NBCSP in the Casablanca-Settat region over the period of January 1, 2018 to December 31, 2021. We did not include 2022 in this evaluation due to concerns about the reliability of data collected that year as the program transitioned to a new health information system. With a 74% urban population, Casablanca-Settat is the largest urbanized region of Morocco. It is situated in the center west part of the country, and is one of twelve administrative regions. In 2018, the region comprised an estimated 7,264,866 inhabitants, representing 20.5% of Morocco’s total population (**Data source**: 2014–2024 Population Projections, Morocco Ministry of Health). The region is comprised of two prefectures (Casablanca and Mohammédia) and seven provinces (Benslimane, Berrechid, El Jadida, Médiouna, Nouaceur, Settat, Sidi Bennour) Fig. 1. Nearly half (49%) of the regional population is concentrated in the prefecture of Casablanca, which is further sub-divided into the following eight districts: Casablanca-Anfa, Al Fida-Mers Sultan, Aïn Sebaâ-Hay Mohammadi, Hay Hassani, Aïn Chock, and Sidi Bernoussi, Ben Msick, and Moulay Rashid [16].

### Organization of the NBCSP in the Casablanca-Settat region

The NBCSP was rolled out in the Casablanca-Settat region in 2011. The program’s early detection activities are integrated into reproductive health services at multiple levels of the Moroccan healthcare system and continue to be gradually scaled up nationally. The NBCSP targets women aged 40 to 69 years as well as women with a family history of breast cancer for biennial screening. Women with a prior diagnosis of breast cancer, however, are excluded from participating in the program [6]. In 2018, the Casablanca-Settat region comprised 1,154,025 eligible women for breast cancer screening. This eligible population increased to 1,190,883 in 2019, 1,226,342 in 2020, and 1,260,386 in 2021 (**Data source**: 2014–2024 Population Projections, Morocco MOH).

**Fig. 2:**
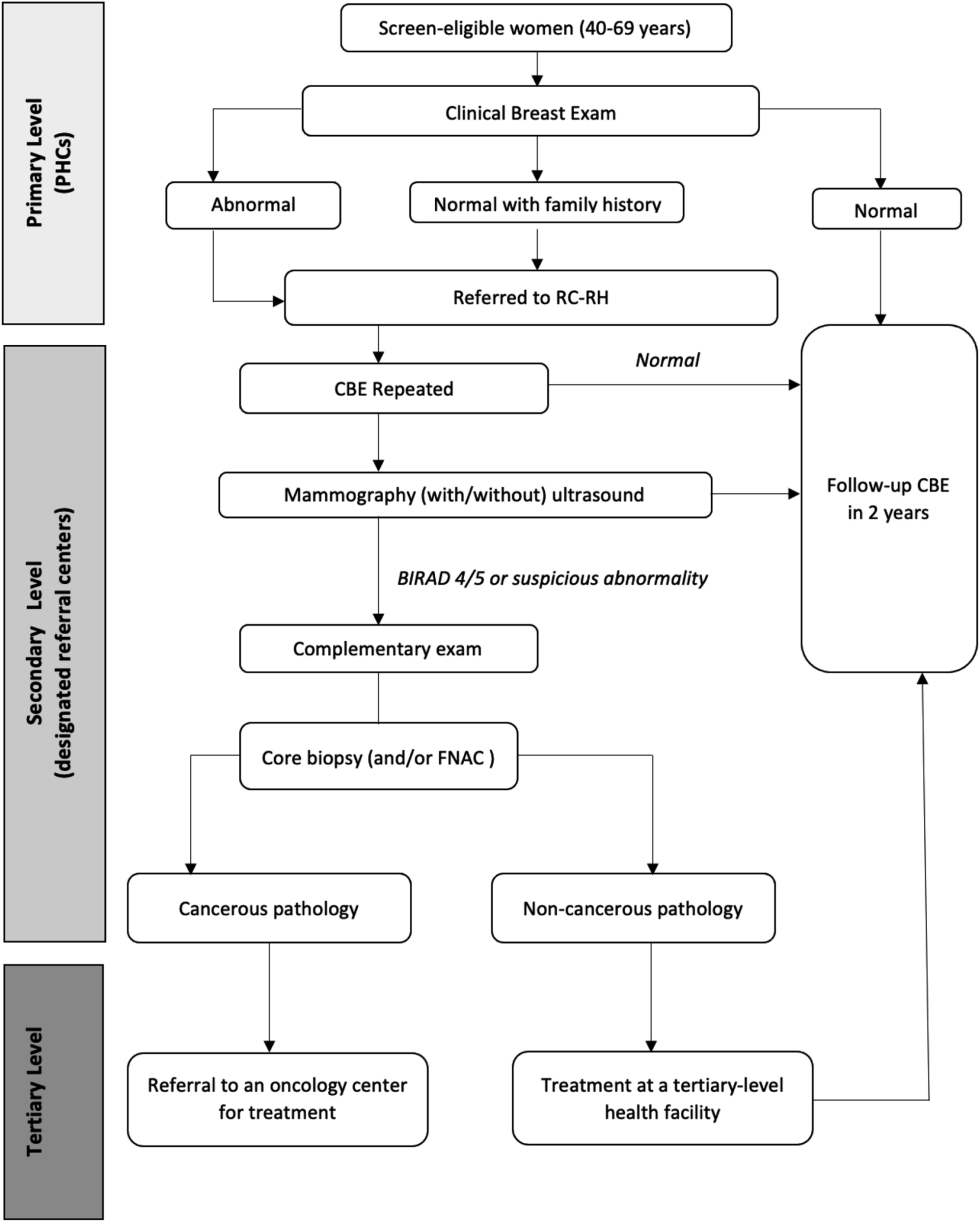
Breast cancer screening algorithm for the NBCSP

Figure 2 presents an algorithm for breast cancer screening within the framework of the NBCSP. Eligible women are screened via CBE at level-I primary health centers (PHCs) by general practitioners, midwives, or trained maternal and child health/family planning nurses. Women with positive (or abnormal) CBE results or those identified as having a family history of breast cancer are then referred to a designated secondary referral center for reproductive health for further diagnostic evaluation. At a secondary referral center, a CBE is repeated by a gynecologist who then requests a mammogram and/or ultrasound in the event of any clinical abnormality of the breast or even an isolated axillary lymphadenopathy. If a suspicious abnormality is identified following radiological exploration of both breasts, a gynecologist performs a core needle biopsy, or by default, a fine needle aspiration cytology (FNAC) procedure on the patient. Biopsy samples are sent to a private pathology lab for examination [6]. All program participants diagnosed with breast cancer are subsequently referred to one of Morocco’s nine regional oncology centers, where they are comprehensively managed [5].

As of January 2018, there were 254 level-I PHCs in the region of Casablanca-Settat [16]. Women screen-detected with breast abnormalities at a level-I PHC from 2018 to 2021 were referred to seven secondary referral centers located in the provinces of El Jadida, Berrechid, and Benslimane, the prefecture of Mohammedia, and the Casablanca districts of Al Fida-Mers Sultan, Aïn Sebaâ-Hay Mohammadi, and Moulay Rashid. The clinical management of all women diagnosed with breast cancer in the Casablanca-Settat region occurs at the University Hospital Ibn Rochd Oncology Center.

### Performance indicators for the NBCSP

This evaluation of the NBCSP is based on a set of key performance indicators and pre-defined standards or goals delineated in Morocco’s national breast cancer screening guideline [6]. Performance indicators analyzed in this study included the following: (1) screening coverage rate (number of women screened at PHCs / eligible population * 100), (2) screening participation rate (number of women screened at PHCs / target population * 100), (3) CBE-positivity rate (number of women with a positive CBE result at PHCs / number of women screened at PHCs * 100), and (4) breast cancer detection rate (number of confirmed breast cancer diagnoses / number of women screened at PHCs * 1000.) The NBCSP aims to achieve a 40% annual screening coverage rate of eligible women in the Casablanca-Settat region, which it defines as its target population [16]. Other objectives of the program are to achieve a 60% screening participation rate among the target population, and to refer 10–13% of screened women for further diagnostic evaluation for a positive CBE result. This standard CBE positivity range was established following a 2009 pilot study in Temera city, Morocco [11].

### Data sources

We extracted aggregated regional data on screening and diagnostic activities under the NBCSP from the MOH’s national health information system for maternal and child health, family planning, and curative care. This information system was established in 2017 to facilitate the reporting of health data and monitoring of health programs. Programmatic data for the NBCSP are manually collected by clinical staff at PHCs and secondary referral centers. Records of women undergoing CBE or diagnostic procedures are maintained in paper-based registers supplied by the program. A written protocol also exists to standardized data collection and program monitoring across all health structures participating in the NBCSP.

Data collected within the framework of the NBCSP are regularly shared between different levels of care and management. PHCs submit monthly performance reports to provincial focal points, with information on the number of women screened and the contact details of all CBE-positive women. These provincial focal points cross-reference the list of CBE-positive women received by PHCs with a list of CBE-positive women undergoing further diagnostic testing at secondary referral centers. They then submit verified performance reports to a regional focal point on a quarterly basis. The regional focal point aggregates these performance data and relays it to the MOH’s program officer in charge of the NBCSP. Site supervisions to a few health structures participating in the NBCSP are also conducted for quality assurance [13].

### Data analysis

We performed descriptive and analytic analyses of NBCSP data for the Casablanca-Settat region. We analyzed annual screening coverage, participation, CBE-positivity, and breast cancer detection rates for the region. We also calculated annual compliance to follow-up visits at secondary referral centers among CBE-positive women. We determined process indicators, notably the number of women screened, CBE positive women, and diagnostic procedures performed (i.e. mammograms, ultrasounds, biopsies) under the NBCSP. These indicators were then stratified by year and sub-region (i.e. province, prefecture, or district). Screening data for the province of Nouaceur, however, was unavailable for 2021.We compared the NBCSP’s screening volume and CBE-positivity rates by year and sub-region using one-way ANOVA and Bonferroni multiple-comparison tests. We also examined annual trends in breast cancer screenings by plotting a graph and computing Pearson’s correlation coefficients to assess how closely these trends were related. Finally, we used the Stata *nptrend* command to statistically test for trends across calendar months. We performed all analyses using Microsoft Excel 2018 and STATA 15.0 [17].

## Results

### Trends in breast cancer screenings in the Casablanca-Settat region

Between 2018 and 2021, a total of 846,692 women were screened for breast cancer across PHCs in the Casablanca-Settat region under the NBCSP. The vast majority of these women were screened for the first time (79.4%, *n* = 672,441), while remaining program participants returned for follow up biennial screening (20.6%, *n* = 174,251). The program recorded its highest screening volume in 2018 with 332,787 women screened, and lowest screening volume in 2020 with 126,889 women screened (Table 1). Overall, the number of women screened under the NBCSP in the Casablanca-Settat region gradually declined over the study period. The annual screening volume in 2019 (*n* = 249,501) was 25.0% below that of 2018. The screening volume further declined in 2020 following the advent of the COVID-19 pandemic. This year saw a 49.1% decrease in total number of screenings compared to the preceding year. This downward trend in breast cancer screenings, however, was reversed in 2021, with the program recording an 8.4% increase in screenings in the Casablanca-Settat region compared to 2020. The average number of screenings under the NBCSP significantly differed by year (*p* = 0.025). Specifically, the average screening volume in 2018 was significantly higher than that of 2020 (*p* < 0.01) and 2021(*p* < 0.01).

**Fig. 3:**
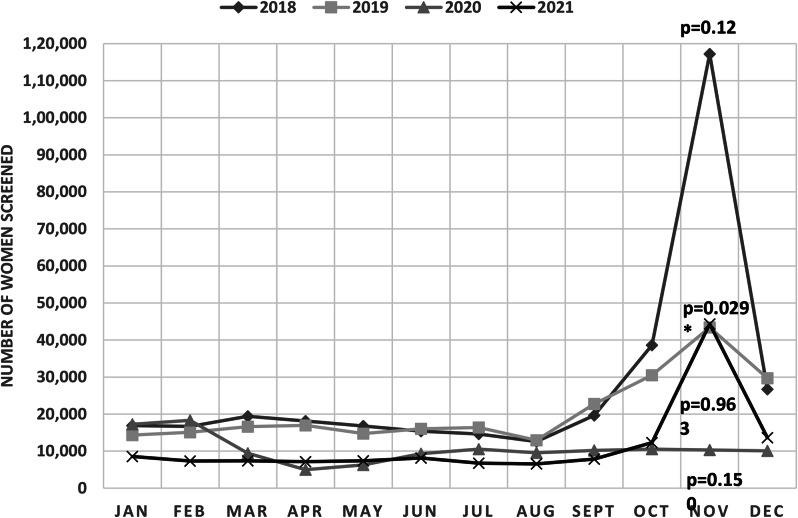
Monthly screenings under the NBCSP in the Casablanca-Settat region (2018-2021). Note: p-values represent the results of nonparametric tests for trends using the *nptrend* command. *p*<0.05 indicates a significant trend in screening across the level of calendar months

Seasonal variations in breast cancer screenings were observed across the region (Fig. 3). Monthly screening volumes were substantially higher between the months of October and December, coinciding with the rollout of a nationwide breast cancer awareness campaign in Morocco [13]. Indeed, more than half of breast cancer screenings performed in 2018 (54.9%) and 2021 (51.1%) were registered over this three-month period. The exception to this seasonal pattern was in 2020 when monthly screenings were highest in January and February (28.0% of total annual screenings). Correlation analyses identified a strong positive correlation between the number of screenings and calendar months in 2019 (*r* = 0.745), but moderate to poor correlations between these two factors in 2018 (*r* = 0.496), 2020 (*r* = -0.353), and 2021 (*r* = 0.504). A significant trend in breast cancer screenings across calendar months was only present in 2019 (*p* = 0.029).

### Referrals and CBE positivity rates

Overall, a total of 21,476 screening participants in the Casablanca-Settat region were referred to a secondary referral center for a positive CBE result (Table 1). CBE positivity rates in the region remained relatively constant over the study period at an average of 2.4% (range: 2.2–2.7%), and did not significantly differ by year (*p* = 0.452). There were, however, significant variations in CBE-positivity rates between different provinces, prefectures, and districts in the Casablanca-Settat region (*p* < 0.01). From 2018 to 2021, the provinces of Settat (0.9%), Berrechid (1.3%), and the Casablanca district of Ben M’sick (1.5%) reported the lowest average CBE positivity rates among women screened under the NBCSP. While the provinces of Benslimane (8.4%), Mediouna (5.2%), and the prefecture of Mohammedia (6.6%) reported the highest average CBE positivity rates among screening participants.

**Table 1:** Total screenings and referrals under the NBCSP in the Casablanca-Settat region (2018–2021)

Province & prefecture of Casablanca-Settat	2018		2019		2020		2021	
	No. of women screened at PHCs	No. (%) of women referred with positive CBEs	No. of women screened at PHCs	No. (%) of women referred with positive CBEs	No. of women screened at PHCs	No. (%) of women referred with positive CBEs	No. of women screened at PHCs	No. (%) of women referred with positive CBEs
Benslimane	7,425	642 (8.6)	4,671	501 (10.7)	2,337	168 (7.2)	2,648	184 (6.9)
Berrechid	37,876	549 (1.4)	35,061	814 (2.3)	19,845	179 (0.9)	25,327	164 (0.6)
El Jadida	42,902	893 (2.1)	25,251	637 (2.5)	12,427	271 (2.2)	17,458	423 (2.4)
Mediouna	5,174	153 (3.0)	3,587	223 (6.2)	1,693	83 (4.9)	1,711	132 (6.8)
Mohammedia	8,432	185 (2.2)	4,569	202 (4.4)	2,148	237 (11.0)	1,192	105 (8.8)
Nouaceur	13,646	702 (5.1)	6,176	245 (4.0)	5,694	207 (3.6)	-------	-------
Settat	31,241	470 (1.5)	25,180	280 (1.1)	10,877	59 (0.5)	7,211	72 (0.6)
Sidi Bennour	12,563	512 (4.1)	6,109	167 (2.7)	3,903	158 (4.0)	4,312	154 (3.6)
Casablanca (by district)								
o Casablanca Anfa	29,267	668 (2.3)	22,987	487 (2.1)	10,325	272 (2.6)	18,256	356 (1.9)
o Al Fida-Mers Sultan	19,928	674 (3.4)	11,016	301 (2.7)	6,217	221 (3.6)	5,656	217 (3.8)
o Aîn-Sebaâ - Hay Mohammadi	27,980	626 (2.2)	23,986	543 (2.3)	13,514	217 (1.6)	9,420	242 (2.6)
o Hay Hassani	20,183	854 (4.2)	7,346	306 (4.2)	2,093	86 (4.1)	6,180	285 (4.6)
o Aîn-Chock	28,921	717 (2.5)	25,755	727 (2.8)	4,538	159 (3.5)	47,40	259 (5.5)
o Sidi Bernoussi	21,342	645 (3.0)	16,634	467 (2.8)	11,661	241 (2.1)	10,264	229 (2.2)
o Ben M’sick	13,828	416 (3.0)	19,405	270 (1.4)	13,455	131 (1.0)	9,801	68 (0.7)
o Moulay Rachid	12,079	309 (2.6)	11,768	417 (3.5)	6,162	126 (2.0)	7,540	169 (2.2)
**Total Regional**	**332**,**787**	**9015 (2.7)**	**249**,**501**	**6587 (2.6)**	**126**,**889**	**2815 (2.2)**	**137**,**410**	**3**,**059 (2.2)**

Note: PHCs = Primary health centers; CBEs = Clinical breast exams

### Screening coverage and participation rates

In 2018, screening coverage of eligible women in the Casablanca-Settat region under the NBCSP was 28.8%. By 2021, the program’s coverage had declined to 10.4% (Table 2). The NBCSP’s average screening coverage from 2018 to 2021was 17.8%. Average screening coverage over the study period was highest in the province of Berrechid (44.0%), and the Casablanca districts of Ben M’sick (28.6%) and Casablanca-Anfa (25.7%). Conversely, average coverage was lowest in the prefecture of Mohammedia (5.9%), and districts of Hay Hassani (8.3%) and Moulay Rachid (9.9%). Screening coverage rates, however, were not found to be significantly different by sub-region (*p* = 0.508). During the study period, only two provinces (Berrechid and El Jadida) attained 40% annual screening coverage of eligible women in 2018. Meanwhile, participation among the NBCSP’s target population in the Casablanca-Settat region reached a high of 72.1% in 2018 and a low of 25.9% in 2020 (Table 2). An annual participation rate of the target population of 60% or higher was only achieved in 2018. Overall, annual participation among the target population from 2018 to 2021 decreased by 44.8%.

**Table 2:** Screening coverage and participation under the NBCSP in the Casablanca-Settat region (2018–2021)

Year	Screen-eligible population (women 40–69 years)	Annual target population	No. of women screened	% of eligible population screened	% of annual target population screened
2018	1,154,025	461,610	332,787	28.8%	72.1%
2019	1,190,883	476,353	249,501	21.0%	52.4%
2020	1,226,342	490,535	126,889	10.4%	25.9%
2021	1,260,386	504,154	137,410	10.9%	27.3%

### Diagnostic activities and breast cancer detection rates

**Fig. 4:**
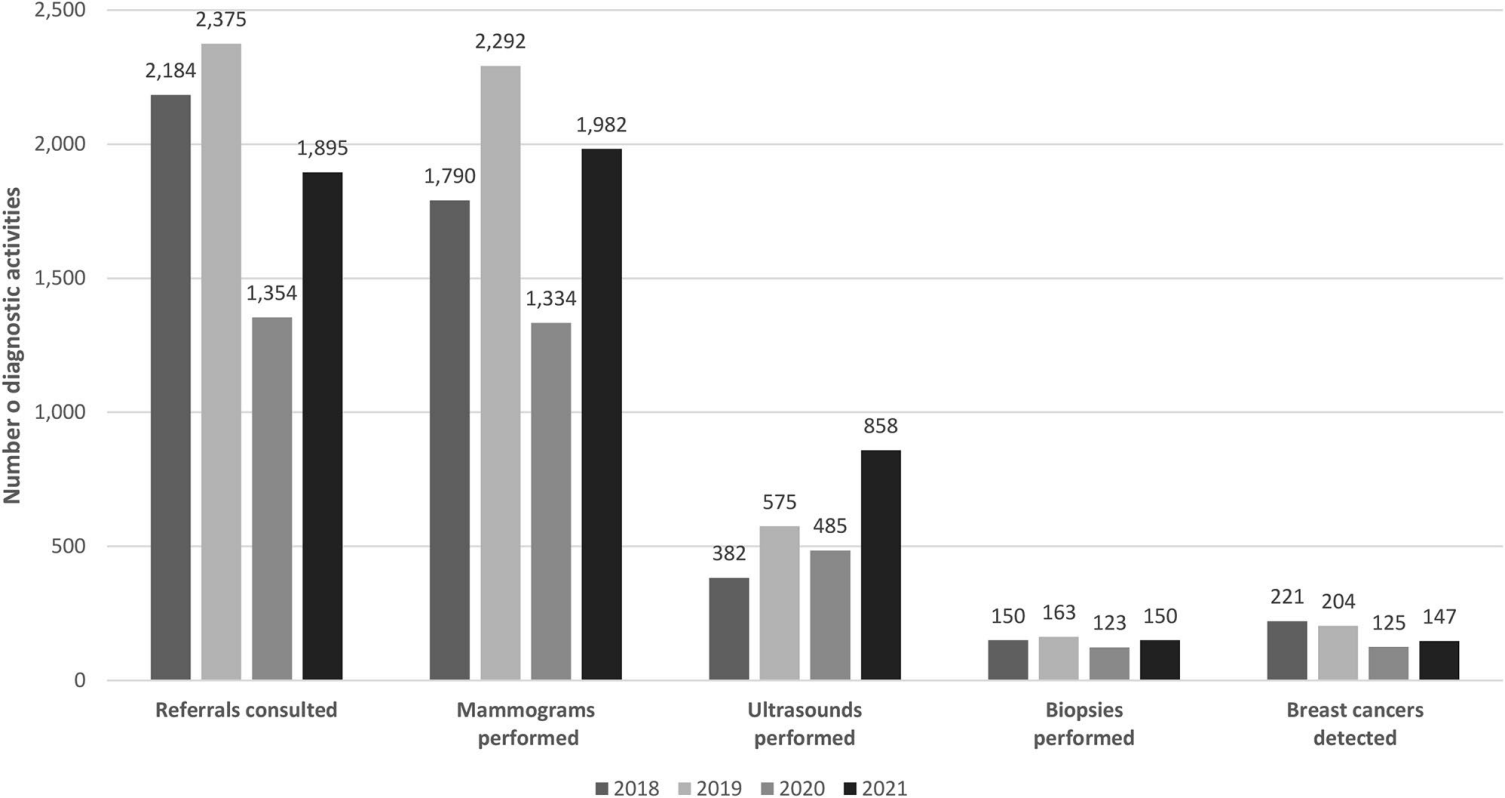
Breast cancer diagnostic activities under the NBCSP in the Casablanca-Settat region (2018-2021)

A little over one-third of (36.4%, *n* = 7,808) of CBE-positive women under the NBCSP sought consultations at designated secondary referral centers in the Casablanca-Settat region. Compliance of CBE-positive women to follow-up diagnostic testing at these facilities more than doubled during the study period, from 24.2% in 2018 to 61.9% in 2021. A total of 7,398 mammograms, 2,300 ultrasounds, and 586 biopsies were performed at secondary referral centers during the study period (Fig. 4). The number of women undergoing mammography at these centers decreased by 86.6% in 2020 compared to 2019. However, a 43.3% increase in mammography procedures was recorded in the subsequent year.

From 2018 to 2021, an average of 8.1% (range: 7.1 − 9.2%) of women seeking consultations at a secondary referral center in the Casablanca-Settat region received a biopsy on site. Additionally, 697 women participating in the NBCSP were diagnosed with breast cancer. The number of breast cancers diagnosed through the program decreased by 38.7% in 2020 compared to 2019. Meanwhile, the NBCSP’s breast cancer detection rate in the Casablanca-Settat region was 0.7 per 1000 women screened in 2018. The detection rate slightly increased to 0.8, 1.0, and 1.1 per 1000 women screened, respectively, from 2019 to 2021. The overall breast cancer detection rate from 2018 to 2021 was 0.8 per 1000 women screened (697/846,692).

## Discussion

The present study evaluated key performance indicators of Morocco’s NBCSP for the Casablanca-Settat region from 2018 to 2021, comparing them against national quality standards. Our findings demonstrate that the screening program fell short of attaining its desired performance benchmarks during the four-year period, notably as it pertains to screening coverage, participation, and CBE positivity rates. Over the study period, screening coverage of eligible women in the Casablanca-Settat region under the NBCSP ranged from 10.4 to 28.9% per year, well below the annual 40% threshold targeted by the Moroccan MOH. We also observed a substantial decline in the program’s screening activities overtime, as annual participation rates in the target population nearly halved over the study period. Annual breast cancer detection rates for the region under the NBSCP were generally low but fairly consistent. While compliance to follow-up diagnostic testing at designated secondary referral centers among CBE-positive women substantially improved over the study period.

Although, the program reported relatively stable annual CBE-positivity rates from 2018 to 2021, at an average of 2.4%, these were considerably lower than the expected standard of 10–13% of screened women being referred for a positive CBE result [6]. There was also notable variability in CBE positivity rates at the sub-regional level, suggesting differences in the quality of the exam provided and a need to standardize CBE training across PHCs in Casablanca-Settat to address quality assurance questions. Annual CBE positivity rates in this study (2.2–2.7%,) were slightly higher than the average CBE positivity rate (1.28%) reported in a long-term screening trial among women 35–64 years in Mumbai, India [18]. Alternatively, the rates reported in our study were substantially lower than the CBE-positivity rate (8.3%) found in an unscreened population of women in rural Indonesia [19].

Prior studies evaluating the NBCSP in other regions of Morocco have similarly reported moderate to low screening coverage of eligible women under the program. Between 2009 and 2011, 36% of eligible women were screened in the pilot program initiated in Temara city [11]. Additionally, between 2012 and 2014, 26% of eligible women participated in the NBCSP in the Meknes-Tafilalt region [12]. Meanwhile, IARC’s national evaluation of the NBCSP uncovered a 36.2% screening coverage rate of eligible women in the Casablanca-Settat region in 2015 [13]. However, the lower screening coverage found in this study compared to IARC’s evaluation can be partially explained by an expansion in the age-eligible screening population for the NBCSP since 2016 [13].

Our findings revealed a sharp decline in screenings under the NBSCP in 2020 (-49.1%), suggesting the COVID-19 pandemic may have had a major impact on women’s screening behaviors in the Casablanca-Settat region. Several studies investigating the impact of the pandemic on utilization of cancer care services have reported similar reductions in breast cancer screenings over the same period. A meta-analysis found a 46.7% decrease in global breast cancer screenings from January to October 2020 [20]. Another systematic review reported that most mammography-based screening programs in North America, Europe, and Asia experienced declines of ≥ 49% in their screening volumes during the early-phase of the pandemic [21]. Although we cannot definitively establish the exact reasons behind the screening decline in Casablanca-Settat region, we speculate that Morocco’s national COVID-19 response may have been a key factor. Confinement measures put in place in mid-March 2020, following the declaration of a state of health emergency, greatly restricted the movement of people and reduced the availability of health services [22]. Routine screening was temporarily postponed, and mass awareness campaigns were suspended [23]. Fear of COVID-19 exposure at medical sites could have additionally influenced women’s decisions not to undergo breast cancer screening [24].

In the post-COVID period of this study, we also found that CBE-positive women in the Casablanca-Settat region were more likely to comply to receiving further diagnostic testing at a designated secondary referral center. Indeed, the proportion of CBE-positive women visiting these centers for diagnosis and treatment more than doubled from 2018 to 2021. In prior evaluations, the need for additional visits to a secondary referral center was identified as a rate limiting step of the NBCSP, one that could lead to poor compliance in diagnostic evaluation [13, 25]. Our results indicate otherwise, since nearly two-thirds of CBE-positive women under the NBCSP in 2021 selected to visit a secondary referral center linked to the program. This change in care-seeking practice is perhaps indicative of a strengthening of referral pathways for breast cancer care within Morocco’s public health system. Alternatively, it may have been a consequence of reduced service provision by the private sector and greater restricted access to referral hospitals during the pandemic [26, 27].

Compared to international benchmarks, annual breast cancer detection rates found in this study were considerably lower than detection rates previously reported from mammography-based screening programs in Europe (5.3/1000) and the United States (5.1/1000) [28, 29]. Moreover, higher detection rates have been reported under CBE-based screening interventions in Cairo, Egypt (3/1000) [30] and in certain sub-Saharan African countries (≤ 3.3/1000) [31]. Our results also found slightly lower breast cancer detection rates in the Casablanca-Settat region during the study period than in prior evaluations of the NBCSP in Meknes-Tafilat(1.2/1,000), Tangier-Tetouan (1.8/1000), and Gharb-Chrarda-Beni Hssen (1.8/1,000) [12, 13]. The NBCSP’s low detection rates may indicate low breast cancer incidence among the eligible population in the region.

As the most urbanized region in Morocco, the Casablanca-Settat region was expected to yield higher breast cancer detection rates than more rural regions of the country due to greater exposure to known breast cancer risk factors [32]. Our results, suggest otherwise. This could be explained by differences in health service utilization patterns across regions. In Morocco, the private sector accounts for 60% of healthcare spending and is heavily concentrated in the Casablanca-Settat and Rabat-Salé-Kénitra regions [33]. Studies have also found that healthcare consumers in the country prefer private providers to public ones [34]. Consequently, breast cancer detection rates for the Casablanca-Settat region may be underestimated by the NBCSP because many CBE-positive women choose to undergo diagnostic testing in the private sector, outside the purview of the program. The high losses to diagnostic-follow-up of abnormal CBEs observed in this study can thus be partially explained by a considerable deviation of patients into the private healthcare sector. Nonetheless, other key patient and health system-related barriers may also be contributing to this loss-to-follow-up, such as fear, stigma, inadequate follow-up planning by healthcare providers, and the non-prioritization of women’s health in the family [35].

Several limitations should be noted in this study. The lack of individual-level data on screening participants did not allow for further analyses of socio-demographic factors associated with screening participation. Furthermore, the NBCSP’s impact on clinical downstaging and survival could not be assessed due to the absence of data sharing mechanisms between the program’s secondary referral centers and tertiary-level hospitals in the Casablanca-Settat region. This deficit does not allow for the tracking of participant’s treatment outcomes and makes it harder to acquire diagnostic results, contributing to the underestimation of the program’s breast cancer detection rates [13].

Our findings highlight the challenges of systematically implementing a national screening program in a middle-income country. Although current data suggest CBE may be the most appropriate screening modality for lower resource settings [30], we found that its uptake is largely dependent on effective mass campaigns as reflected by drastic seasonal variations in screening patterns. Awareness campaigns should be tailored to the local context and routinely evaluated to ensure fidelity, reach, and efficacy in breast cancer knowledge transfer. Specific guidelines for CBE screening and training protocols should also be formulated to standardize the quality of CBE exams across public health facilities. Results from this study also underscore the need to establish well-functioning information systems for successful program monitoring and evaluation. In Morocco, it is crucial that the NBCSP establish linkages to regional oncology centers to better ascertain the proportion of breast cancers detected via the program and assess the shift in stage distribution achieved among screening participants. Collecting individual data on screening participants will be critical to informing strategies and optimizing the effectiveness of the NBCSP in the Casablanca-Settat region.

## Conclusions

Key performance indicators of Morocco’s NBCSP for the Casablanca-Settat region were evaluated over a four-year period. Screening coverage across the region was largely below the desired threshold, and participation among the target population substantially declined from 2018 to 2021. In particular, a sharp drop in breast cancer screenings was recorded in 2020, suggesting the COVID-19 pandemic had a major impact on women’s screening behaviors in the Casablanca-Settat region. Other performance indicators of the NBCSP were not impacted by the pandemic. Annual CBE positivity rates remained relatively constant throughout the study period, though far below the expected standard level. Annual breast cancer detection rates remained generally low over the study period, however compliance to further diagnostic testing at designated secondary referral centers substantially improved among CBE-positive women. Results from this study underscore the need for additional interventions to increase breast cancer screening participation in the Casablanca-Settat region and address gaps in breast cancer care referral pathways within the public sector.

## Data Availability

The datasets used and/or analyzed during the current study are available from the corresponding author on reasonable request.

## Abbreviations

CBE: Clinical breast examination
FNAC: Fine needle aspiration cytology
LMICs: Low and middle-income countries
NBCSP: National breast cancer screening program
MOH: Ministry of Health
PHCs: Primary health centers
WHO: World Health Organization
